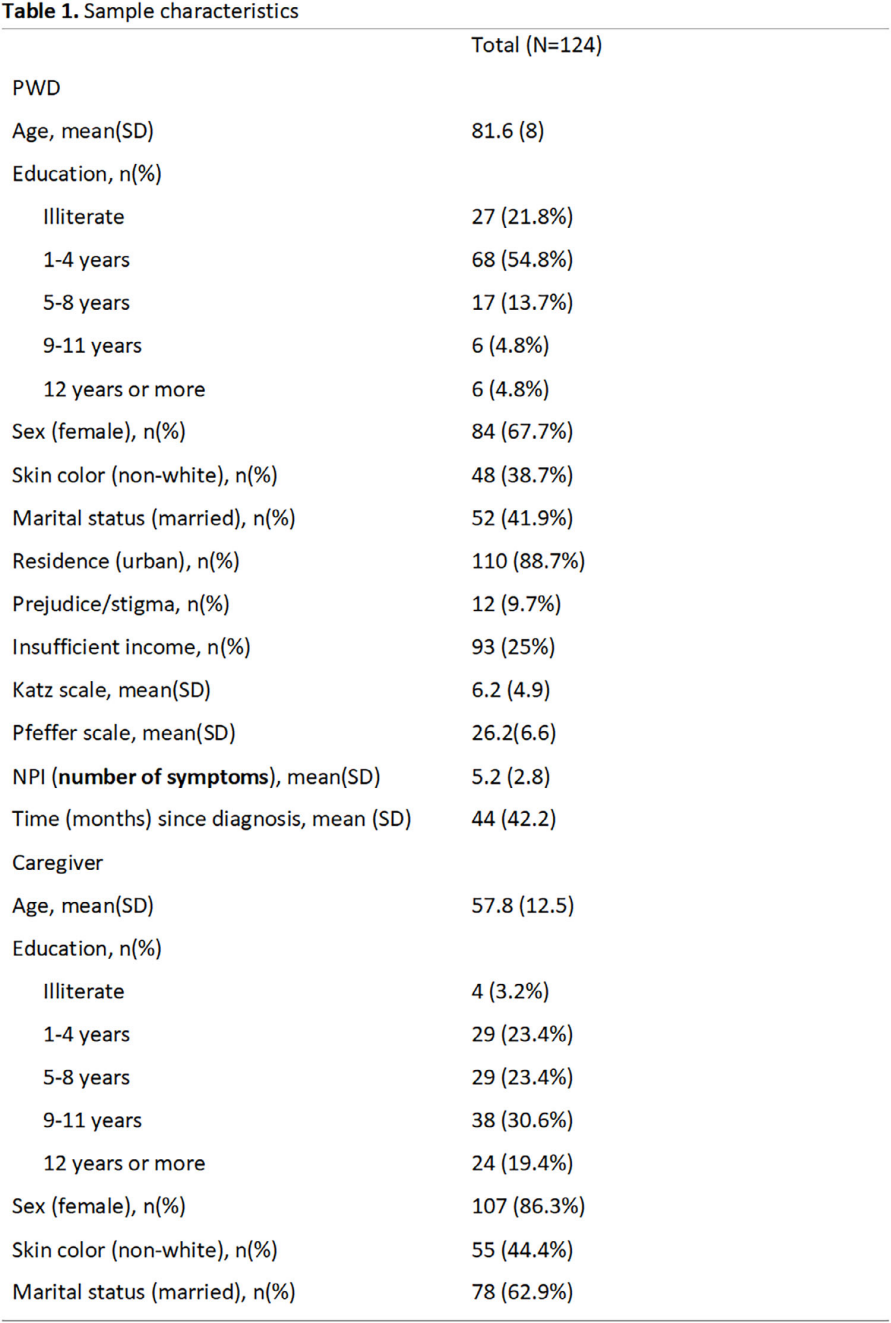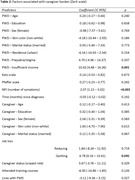# Factors associated with dementia caregiver burden across multiple Brazilian regions

**DOI:** 10.1002/alz.092373

**Published:** 2025-01-09

**Authors:** Matheus Ghossain Barbosa, Marcos Antonio Costa Ferreira de Macedo, Fabiana A. F. da Mata, Ari Alex Ramos, Haliton Alves de Oliveira Júnior, Laiss Bertola, Cleusa P. Ferri

**Affiliations:** ^1^ Universidade Federal de São Paulo (UNIFESP), São Paulo, São Paulo/SP Brazil; ^2^ Universidade Federal de São Paulo (UNIFESP), São Paulo, São Paulo Brazil; ^3^ Hospital Alemão Oswaldo Cruz, São Paulo, São Paulo Brazil

## Abstract

**Background:**

The number of people with dementia (PWD) is increasing worldwide, and especially in low‐ and middle‐income countries (LMIC). Dementia’s burden extends beyond mortality and healthcare costs. In LMIC, dementia indirect costs are proportionally higher. In Brazil two thirds of the total dementia costs come from informal caregiving, mainly provided by women who quit their jobs to act as a carer for a relative.

**Method:**

The ReNaDe project assessed dementia’s cost, unmet needs, and investment. We recruited 140 PWD‐caregiver dyads in 17 cities across the 5 Brazilian regions. PWD were selected through their public healthcare system medical charts, we included PWD 60 years and older at any dementia severity. For this analysis we evaluated the total score of the Zarit Burden Interview (ZBI) as our main outcome and used linear regression to test potential associated factors (sociodemographic, behavioral and clinical).

**Result:**

We included 124 participants with complete data. More neuropsychiatric symptoms in the Neuropsychiatric Inventory (β = 2.07, p = 0.000), having an insufficient income to cover all household costs (β = 10.42, p = 0.001) and quitting, but not reducing, a job (β = 6.78, p = 0.045) were associated with higher burden, while having a replacement caregiver (β = ‐9.87, p = 0.001) was associated with lower burden. The caregiver or patient’s sex, age, marital status, skin color, level of education and place of residence (urban or rural) were not associated with burden level. Furthermore, the length of the caregiving role, previous training courses, the length of the dementia diagnosis, whether the caregiver is paid, lives with the patient or reports the dyad as being a target of prejudice were not associated with level of burden. Finally, the patient’s dependence level as assessed by the Katz and the Pfeffer scales were also not associated with burden.

**Conclusion:**

Most challenges to caregiving don’t appear to directly affect level of burden. More behavioral symptoms are associated with more burden, as is losing a job/income and being the only available caregiver. Caregiver support policies should focus on providing financial support and/or care alternatives to allow for free time, as well as training on how to deal with behavioral issues.